# The translation into Portuguese of the 11^th^ International Statistical Classification of Diseases and Related Health Problems (ICD-11)

**DOI:** 10.1590/1980-549720230043.2

**Published:** 2023-10-09

**Authors:** Elisabeth Barboza França, Daisy Maria Xavier de Abreu, Fatima Marinho, Giovanny Vinicius Araújo de França, Juan Córtez-Escalante, Ada Ávila Assunção, Grupo de tradutores da CID-11 para o português do Brasil

**Affiliations:** Universidade Federal de Minas Gerais, Faculdade de Medicina, Programa de Pós-Graduação em Saúde Pública – Belo Horizonte (MG), Brasil.; Universidade Federal de Minas Gerais, Faculdade de Medicina – Belo Horizonte (MG), Brasil.; Vital Strategies – São Paulo (SP), Brasil.; Ministério da Saúde, Secretaria de Vigilância em Saúde – Brasília (DF), Brasil.; Organização Pan-Americana da Saúde, Organização Mundial da Saúde, Unidade Técnica de Vigilância, Preparação e Resposta à Emergências e Desastres – Brasília (DF) – Brasil.

**Keywords:** International statistical classification of diseases and related health problems, CID-11, Health information systems, Vital statistics, Translations, Classificação estatística internacional de doenças e problemas relacionados à saúde. CID-11, Sistemas de informação em saúde, Estatísticas vitais, Traduções

## Abstract

The 11^th^ International Statistical Classification of Diseases and Related Health Problems (ICD-11) represents an advance in the focus on knowledge and new disease approaches. The ICD is used for different practical purposes, enabling assessment of progress in the global health agenda, resource allocation, patient safety, health care qualification, and health insurance reimbursement. It is entirely digital, with technological resources that allow periodic updating. In early 2022, ICD-11 entered into official force, having been made available in several official ICD languages such as Arabic, Chinese, Spanish, French, and English. The translation process into Brazilian Portuguese, coordinated by the Federal University of Minas Gerais (UFMG), with support from the Brazilian Ministry of Health (MS) and PAHO/WHO, is presented here. The work was carried out in three stages between August 2021 and December 2022 by translators with different backgrounds: medical specialists (49), physiotherapists (1), pharmacologists (1), and dentists (1). This methodological article aims to broaden the discussion of perspectives on implementing the ICD-11 in Brazil and build an opportunity for its adaptation and use by other Portuguese-speaking countries.

## RESUMO

A 11^a^ Classificação Estatística Internacional de Doenças e Problemas Relacionados à Saúde representa um avanço no enfoque do conhecimento e em novas abordagens das doenças. A Classificação Estatística Internacional de Doenças e Problemas Relacionados à Saúde é utilizada para diferentes finalidades práticas, possibilitando avaliação do avanço da agenda de saúde global, alocação de recursos, segurança do paciente, qualificação da assistência à saúde e reembolso de seguros de saúde. É inteiramente digital, com recursos tecnológicos que permitem sua atualização periódica. No início de 2022, a 11^a^ Classificação Estatística Internacional de Doenças e Problemas Relacionados à Saúde entrou em vigência oficial, tendo sido disponibilizada em vários de seus idiomas oficiais, como o árabe, chinês, espanhol, francês e inglês. Apresenta-se aqui o processo de tradução para a língua portuguesa em uso no Brasil, coordenado pela Universidade Federal de Minas Gerais, com apoio do Ministério da Saúde do Brasil e da Organização Pan-Americana da Saúde/Organização Mundial da Saúde. O trabalho foi realizado em três etapas entre agosto de 2021 e dezembro de 2022 por tradutores com diferentes formações: médicos especialistas (49), fisioterapeuta (1), farmacologista (1) e odontologista (1). Com este artigo metodológico, almeja-se ampliar a discussão de perspectivas para implementação da 11^a^ Classificação Estatística Internacional de Doenças e Problemas Relacionados à Saúde no Brasil e construir uma oportunidade para sua adaptação e uso por outros países de língua oficial portuguesa.

## INTRODUCTION

The production of statistics on the frequency of diseases and causes of death in the population requires the use of a standardized classification to define the nomenclature and grouping according to certain criteria. The first international classification for causes of death was approved in 1893 by the International Institute of Statistics, known as the Bertillon Classification. On the recommendation of the American Public Health Association, this classification began to be used from 1898 onward in the statistical services of Mexico, Canada, and the United States, and was later disseminated to several countries. Since its adoption, it has been defined and agreed on the need for periodic reviews, which have been the responsibility of the International Statistical Institute (first, second, and third reviews) and the French government (fourth and fifth reviews)^1^.

With the sixth revision, in 1948, the World Health Organization (WHO) assumed responsibility for the elaboration and periodic revisions of the international classification, in order to reflect advances in public health and medical sciences. In this classification, there were significant changes, such as the incorporation of diseases and reasons for consultation for health problems, with the name being changed to the International Statistical Classification of Diseases and Causes of Death, known simply by the acronym ICD. This characteristic explains the significant increase in the number of categories (from 200 in ICD-4 and ICD-5 to 1,010 in ICD-6). It is important to note that, in the sixth revision, standardized rules were included for selecting the underlying cause of death and definitions for use in health statistics, particularly the definitions of vital events and those for morbidity^2^. 

In the International Conference for the Tenth Revision of 1989, the ICD-10 was approved, as well as the strategy of composing a family of integrated classifications. Used alone or together, these classifications contribute to providing health or health-related information. The name family is appropriate because it refers to the reference classifications, as well as to derived classifications and related classifications, known as World Health Organization – Family of International Health Related Classifications (WHO-FIC). The first to be included was the International Classification of Functioning, Disability and Health (ICF), which is considered a reference along with the ICD^3,4^. Together, these classifications are fundamental tools to address the multiple dimensions that involve human health, including the characteristics of services that assist populations^4,5^.

In Brazil, the Brazilian Center for Disease Classification (CBCD) was created in 1976 through an agreement between the University of São Paulo, the Pan American Health Organization/World Health Organization (PAHO/WHO) and the Ministry of Health. It was responsible for the translation into Portuguese of volumes 1, 2, and 3 of ICD-10 in the 1990s, to compose the base of codes used in the country since 1996 in all information on mortality and several on morbidity, being designated in 2001 “WHO Collaborating Center for the Family of Classifications in Portuguese”. Its activities ended in March 2016^1,3,6^, being absorbed by the Ministry of Health^7^. In 2021, the process began to designate the Ministry of Health as a WHO Collaborating Center, a fundamental step given the importance of Brazil in the Family of International Classifications (FIC) network^8,9^. 

In 2007, WHO began the production of ICD-11 with the involvement of a wide range of physicians, researchers, and technicians specialized in health systems, among other professionals, as well as the general public. After more than a decade in the making, the 11^th^ version of the ICD was approved at the 72^nd^ World Health Assembly in May 2019, that is, almost 30 years since the approval of ICD-10^10^. 

The structure of ICD-11 is sophisticated, as it classifies events that express the related processes of becoming ill and dying, assigning them codes that allow capturing clinical characteristics with a high degree of specificity and enabling their summarization into clusters for different purposes. Changes in content reflect the advancement of knowledge in medicine, with design and structure consistent with the networked digital age. These are the most important differences between ICD-10 and ICD-11. The latter is characterized by not being just a simple revision of the former, becoming in fact a different and powerful health information system^11^. It is structured to serve multiple purposes, from supporting doctors on the front line with other health professionals to developing policies to intervene in health realities^4,10,12^.

The original version of ICD-11 was prepared in English. The language was considered the “initial unfavorable aspect” for its implementation in Brazil, given that the entire process of translation, adaptation, revision, and implementation into a new language takes time^13^. In early 2022, ICD-11 entered into official force, having been made available in several official languages of the ICD, such as Arabic, Chinese, Spanish, French, English^14^, and currently also Russian (available on the website icd.who.int/en). Translation into the country’s language is considered the priority step for its implementation^10^.

In Brazil, the Technical Advisory Board for the Management of the Family of International Classifications (*Câmara Técnica Assessora para a Gestão da Família de Classificações Internacionais* – CTA BR-FIC), was established in March 2021, coordinated by the Department of Epidemiological Analysis and Surveillance of Noncommunicable Diseases (*Departamento de Análise Epidemiológica e Vigilância de Doenças Não Transmissíveis* – DAENT) of the Secretariat for Surveillance and Environment (*Secretaria de Vigilância e Ambiente* – SVSA) of the Ministry of Health^5^. Universidade Federal de Minas Gerais (UFMG) was invited by CTA BR-FIC to undertake the translation of ICD-11 into Portuguese^15^. Work began in August 2021, also with the support of PAHO/WHO. 

As expected, in the translation process, impasses and dilemmas arose that required decision-making based on the state of the art, clinical practice, the culture of the professions, the guidelines and the structure of current health systems. This article seeks to present the main changes introduced in the 11^th^ revision of the ICD and how the process of translating this version into the Portuguese language in use in Brazil was carried out. The aim is to build an opportunity for health professionals, society in general and managers responsible for disseminating the ICD-11 in Brazilian Portuguese to learn about the advances of this new version and the challenges for its implementation.

### Some of the many ICD-11 innovations

Innovations are at the heart of the new ICD version, which integrates all its content into a multidimensional structure of entities called Foundation, Fundamental Component or Fundament, comprehensive and connected with online platforms. Entities represent the basic unit of ICD-11 and relate to diseases, disorders, injuries, external causes, signs and symptoms, functional descriptions, interventions, and extension codes. They have a unique identifier, called Unique Resource Identifier (URI), which represents a fundamental support for digital communication^4,11^. 

Lists with mutually exclusive categories, so-called tabular lists, are derived from the Foundation/Fundament, such as the ICD-11 Tabular List for Mortality and Morbidity Statistics (ICD-11 MMS), available in the browser. This online application for accessing and using ICD-11 corresponds to volume 1 of ICD-10 and it presents the 26 chapters and the two supplementary sections that make up ICD-11, with all the subdivisions into blocks, categories, and subcategories. Several chapters are unpublished, two of them as a result of chapter III of ICD-10, in addition to the updated contents and structure changes that permeate the entire list. Attention is drawn to the relocation of strokes from the chapter on cardiovascular diseases to the chapter on diseases of the nervous system^4^.

The ICD-11 tabular list uses a hierarchical structure based on the “family tree” concept to group related entities, called parent-child entities. Parental entities (parents) are at the highest level in the hierarchy, and the child and siblings’ entities provide greater detail when needed. For example, typhoid fever represents a child entity of intestinal bacterial infections and is at the same hierarchical level as the sibling entity of paratyphoid fever and other *Salmonella* infections. An important innovation refers to the fact that an entity can have one or more parents, being presented more than once in the classification set, with emphasis on the primary parent. Thus, cerebrovascular disease has its primary parent in the chapter on diseases of the nervous system and also appears in the chapter on cardiovascular diseases, in the latter case with an indication (lighter colored letters) that its primary location is in another chapter^4,16^.

Another electronic application available is the Coding Tool, for accessing the ICD-11 codes, associating the name of the disease or term with related codes. Functioning as an alphabetical index, it lists approximately 120,000 clinical terms and would correspond to volume 3 of ICD-10. This tool represented a powerful technological advance for the codification of diseases and causes of death, as it allows searching all classification terms as well as accessing additional information, such as the hierarchy of entities in groups and categories. Easy to use, it enables an immediate search with greater accuracy for the appropriate code, through the diagnosis or the terminology used by the user. Its digital structure can be inserted into local digital systems, using the WHO web or offline version^4,11^.

For coding, alphanumeric codes of up to six characters are used in the translation of diagnoses of diseases and other health problems to enable storage, retrieval, and data analysis. The codes are organized from 1A00.00 to ZZ9Z.ZZ and are called stem codes. These are considered highly relevant and may appear isolated in the tabular list. They are composed of letters and numbers, and the first character indicates the chapter. Chapters 1 to 9 start with a number (from 01 to 09), and the following chapters start with a letter, from A to R. The letters O and I are not used because the spelling is similar to that of the numerals zero and one^4^. 

Assigning multiple codes to describe a diagnosis represents a notable innovation from ICD-11. A code cluster occurs in the case of two or more stem codes with various clinical concepts, or in the case of specific details captured in so-called extension codes. There are more than 20 thousand extension codes. The purpose of these supplementary codes, which should never be used alone and begin with the letter X, is to allow greater specificity and recording of additional information, such as severity scale, temporality or life cycle, anatomical and histopathological aspects, lesion characteristics, external causes, occupational characterizations, medications, and other substances, among other elements. This conception, presented by the concept of post-coordination, aims to highlight the complexity of the causal link when, for example, a disease or condition mentioned in the death certificate is related to another disease or condition equally declared^4,11^. This approach, based on the multidimensional paradigm, was designed to extrapolate the record based exclusively on clinical knowledge so that it reproduces knowledge in the field of epidemiology.

In short, ICD-11 facilitates access to terms and related coding, prepared in line with scientific advances and new paradigms in health care. Attention is drawn to the potential for quick and complete registration, made possible by the increased use of electronic tools.

### The process of translating ICD-11 into Brazilian Portuguese

To coordinate the process of translating ICD-11 into Portuguese, the UFMG team initially appropriated the instructional and related materials available on the WHO website. This enabled the necessary improvement to conduct the process, with emphasis on the reference guide^4^. *Pari passu* institutional events promoted by the Ministry of Health of Brazil, by PAHO/WHO and by institutions from other countries on the new contents of ICD-11, such as sexual health and aging, were valuable opportunities for the coordination of the process. In addition, the dialogue with PAHO/WHO professionals who were in charge of the translation into Spanish signaled fundamental elements for the organization of the methodological strategy that was adopted.

The process of translating the terms, between titles of the codes and descriptions of the diseases in the 26 chapters and in the two sections of ICD-11, was carried out in three distinct periods, given the contingencies of the support of the financing institutions: 1^st^ stage — August to November 2021 (PAHO/WHO funding), 2^nd^ stage — March to June 2022 (Ministry of Health funding), and 3^rd^ stage — September to December 2022 (PAHO/WHO funding). The alternative for the participation of translators available in the communication market was initially evaluated, but it did not prove to be viable, considering the complexity and scope of the content that covers most medical specialties, as well as the knowledge and practices of other health areas, such as pharmacology, dentistry, and sexual health.

Academic insertion, mastery of the English language and experience in clinical practice related to specific chapters were the criteria to compose the group of translators, with training in cardiology, endocrinology, dermatology, gastroenterology, gynecology, hematology, family medicine, neonatology, neurology, obstetrics, ophthalmology, otorhinolaryngology, pathology, pediatrics, pulmonology, psychiatry, rheumatology, among other specialties. Professionals with different backgrounds participated: medical specialists (48), physiotherapist (1), pharmacologist (1), and dentist (1), most of them university professors. A specialist epidemiologist was responsible for translating the Interface.

Once the groups of translators were defined, a short training program on the expansion of ICD-11 in relation to ICD-10 was chosen. In the course of workshops and similar activities, the bases of the 11^th^ revision were addressed, as well as the principles of coding and structuring of chapters and supplementary sections. Training was carried out for the proper handling of the electronic platform, called the maintenance platform (or orange platform), in which translation into other languages is carried out, as well as the inclusion of comments or proposals on ICD-11. This activity satisfied the needs in this context, given the support of a professional specialized in information technology. This availability ensured navigation on the electronic platform from registration to the actual translation and revision. The procedures indicated by the coordination were followed, adapted, and improved throughout the translation process, which lasted 17 months in total.

The translator specialists used a variety of sources of consultation, with priority being given to ICD-10, translated into Portuguese in the 1990s by CBCD, trying whenever possible to adopt the terms of that version. In addition to this, other references were included, such as:

- Technical-scientific publications;
- Abstract or text available in Portuguese and English: SciELO, Virtual Health Library (*Biblioteca Virtual em Saúde* – BVS MS);
- Health Sciences Descriptors (*Descritores em Ciências da Saúde* – DeCS), from the Latin American and Caribbean Center on Health Sciences Information (BIREME) (different languages);
- Specialist societies;
- Dictionaries: English-Portuguese and Portuguese-English, Houaiss Dictionary of the Portuguese Language, Webster’s Student Dictionary of the English Language, among others;
- Spanish version of ICD-11 available on the platform;
- Online translation tools: Reverse Translation, Linguee – English-Portuguese dictionary and translation search engine;
- Sites of a technical and academic nature: Atlas of human anatomy, Anvisa, Orphanet (rare diseases and orphan drugs), etc.

Specific instructions were prepared to guide the insertion of comments related to difficulties and decisions taken in the face of dilemmas in the development of the work. It is worth highlighting the relevance of the “comments” field on the translation platform, a device that is part of the electronic structure of ICD-11, intended for adjustments in order to safeguard the semantic contents. The support and planning team was responsible for evaluating the set of comments recorded by the translators and indicating the pertinence of developing and deepening some of them in the proposal format, in case incompleteness or inadequacies were found in the content of the original version in English.

Admitting the need for standardization of common terms to be translated, the strategy was to have a commission of senior medical specialists in information systems and in the ICD itself. The commission’s mission was to draw up agreements to translate recurrent acronyms, expressions, acronyms or abbreviations, among other terms that are part of the text of ICD-11. This commission also represented an important support for the coordination in the discussion of translation impasses that occurred in the first stage of the work process. Examples of so-called translation agreements are shown in Chart 1.

**Chart 1.** Example of translation agreement.

Letter	Term in English	Translation
G	G	
H	H	
	Harmful	nocivo
I	I	
	Injury	lesão ou traumatismo
	Ill-defined	mal definido
J	J	
L	L	
M	M	
	Manifestation	manifestação
N	N	
	NOS (not otherwise specified)	SOE (sem outra especificação)
	NEC (not elsewhere classified)	NCOP (não classificado em outra parte)
O		
P		
Q		
R		
S	S	
	Self-harm	autoinfligidas/autoagressão
	Severe	grave
T	T	
	top level blocks	agrupamentos de níveis superiores
U	U	
	Unspecified	não especificado (a)

### Considerations on the ICD-11 translation process in Brazil

**Figure 1.**
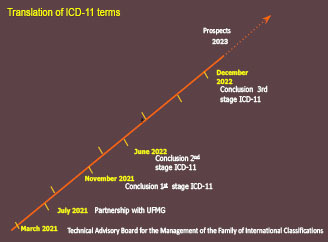
Flowchart of the translation steps of the 11^th^ International Statistical Classification of Diseases and Related Health Problems into Brazilian Portuguese.

More than 1.2 million terms were translated, including code titles and category descriptions of 26 chapters and two supplementary sections, between August 2021 and December 2022. In the first stage, 17 chapters and one supplementary section were translated; in the second, there were seven chapters; and in the third, the last two chapters and supplementary section. Figure 1 and Chart 2 present the completion period of each stage of the translation process and the estimated number of terms translated per chapter and section.

**Chart 2.** Initially estimated number of terms to be translated per chapter and section of the 11^th^ International Statistical Classification of Diseases and Related Health Problems.

ICD-11 chapters for translation	No. of terms
1. Certain infectious or parasitic diseases	81,695
2. Neoplasms	67,619
3. Diseases of the blood or blood-forming organs	23,474
4. Diseases of the immune system	19,378
5. Endocrine, nutritional or metabolic diseases	69,095
6. Mental, behavioural or neurodevelopmental disorders	99,735
7. Sleep-wake disorders	6,950
8. Diseases of the nervous system	80,933
9. Diseases of the visual system	41,336
10. Diseases of the ear or mastoid process	6,054
11. Diseases of the circulatory system	41,262
12. Diseases of the respiratory system	21,869
13. Diseases of the digestive system	50,463
14. Diseases of the skin	65,164
15. Diseases of the musculoskeletal system or connective tissue	38,895
16. Diseases of the genitourinary system	36,291
17. Conditions related to sexual health	3,189
18. Pregnancy, childbirth or the puerperium	18,861
19. Certain conditions originating in the perinatal period	26,848
20. Developmental anomalies	112,339
21. Symptoms, signs or clinical findings, not elsewhere classified	41,515
22. Injury, poisoning or certain other consequences of external causes	53,317
23. External causes of morbidity or mortality	66,895
24. Factors influencing health status or contact with health services	23,062
25. Codes for special purposes	401
26. Supplementary Chapter Traditional Medicine Conditions - Module I	23,711
V. Supplementary section for functioning assessment	18,194
X. Extension codes	86,247
Total terms	1,224,792

Although the feasibility of the deadline defined by the requesting institutions represented a huge challenge for the work to be carried out, the technical competence and responsibility of the specialists involved ensured the completion in time and the quality of the now finalized translated version. The usability of the electronic translation platform, which made it possible to enter the translated terms directly on the website while reading the original text, was a great facilitator of the work. Knowledge of the infrastructure and functioning of health information systems in Brazil and the accumulated experience of public health professionals and highly qualified coding technicians undoubtedly strengthened the translation process presented above.

The insertion of specialist translators in the complex universe of ICD-11 was permeated with surprises, discoveries, and challenges that motivated the work. In this scenario, however, the barriers encountered were seen as steps toward the consolidation of a consistent and robust classification system. The standardization of some terms and operations common to the translation process proved to be useful to optimize the work. Thus, during its development, new agreements were created to guide the translation of terms that are frequent in more than one chapter, observing the specificities of each area. Production of instructions and agreements, on the one hand, doubts and discoveries, on the other, underpinned the dynamic nature of the process of translating ICD-11 into Portuguese. In summary, groups of translators and support and planning staff worked interactively.

For the near future, revisions and adjustments of the finalized version for the current Portuguese language in other countries are planned. The result of this process will be essential to improve the version on screen before its availability scheduled for the end of 2023. This is the first stage of the ICD-11 implementation process, and, for this, in accordance with WHO indications^10^, it is essential to have a national center for technical reference and management of the implementation of ICD-11, which is also responsible for translating its frequent updates. Organizing, adjusting, and maintaining existing classification systems during the transition to ICD-11, assessing the technological infrastructure and developing training programs for coding professionals, identifying possible impacts on existing statistical systems, including and training physicians and managers in a network for sustaining and disseminating are other key interventions in the ICD-11 implementation process in the country. Digital Health has demanded new impulses in the country, in which ICD-11, with its new structure, will improve the generation of more accurate statistics.

The availability of an official version of ICD-11 for the Portuguese language will allow its implementation in Portuguese-speaking countries, contributing to the continuous qualification of health information systems in these countries. To absorb the advances of ICD-11, efficient structuring actions will be necessary in its implementation, especially with regard to the characteristics and structures of the national systems. It will be necessary to meet local needs regarding access to technology and health training, considering the demands of different professionals, whether they are in the management of systems or inserted in routine clinical practice or, even, inserted in the production of statistical information, among other spheres of activity in the field of public health.
